# Multicenter phase ii study of a combination of cyclosporine a, methotrexate and mycophenolate mofetil for GVHD prophylaxis: results of the Chinese Bone Marrow Transplant Cooperative Group (CBMTCG)

**DOI:** 10.1186/s13045-014-0059-3

**Published:** 2014-08-21

**Authors:** Yong-rong Lai, Yu-hong Chen, Deng-ming Hu, Ming Jiang, Qi-fa Liu, Lin Liu, Jian Hou, Paul Schwarzenberger, Qiao-chuan Li, Zhong-ming Zhang, Kai-yan Liu, Xiao-jun Huang

**Affiliations:** 1 Department of Hematology, the First Affiliated Hospital of Guangxi Medical University, Nanning, Guangxi, China; 2 Beijing Key Laboratory of Hematopoietic Stem Cell Transplantation, Peking University People’s Hospital, Peking University Institute of Hematology, Beijing, China; 3 Department of Hematology, Kunming General Hospital of Chengdu Command, Kunming, China; 4 Department of Hematology, the First Affiliated Hospital of Xinjiang Medical University, Wulumuqi, China; 5 Department of Hematology, Nanfang Hospital Southern Medical University, Guangzhou, China; 6 Department of Hematology, First Affiliated Hospital Chongqing Medical University, Chongqing, China; 7 Department of Hematology, Changzheng Hospital, the Second Military Medical University, Shanghai, China; 8 University of South Alabama, Mobile, Alabama, USA; 9 China Peking-Tsinghua Center for Life Sciences, Beijing, China

**Keywords:** Allogeneic hematopoietic stem cell transplantation (allo-HSCT), Graft-versus-host disease (GVHD) prophylaxis, Mycophenolate mofetil (MMF)

## Abstract

**Background:**

Improvement of current GVHD prophylactic therapies remains an important goal in the allo-HSCT. We have described a novel prophylaxis regimen in a single institution trial. The Chinese Bone Marrow Transplant Cooperative Group (CBMTCG) initiated a phase II multicenter study.

**Methods:**

The study was designed as a prospective, single arm phase II open-label, multicenter clinical trial. The primary endpoint was improvement of aGVHD by 25% over historical control (40%) in Chinese patients. 508 patients were enrolled. All of the patients received cyclosporine A (CsA), methotrexate (MTX) and mycophenolate mofetil (MMF) (0.5-1.0 g daily for 30 days) as GVHD prophylaxis regimen.

**Results:**

The primary endpoint was met with cumulative incidences of grades 2 to 4 and grades 3 to 4 aGVHD of 23.2% and 10.3%, respectively. Incidence for cGVHD was 67.4%. The non-relapse mortality (NRM) rate was 18.4% at 2 years. The probabilities of leukemia free survival (LFS) for non-advanced stage and advanced stage patients at 2 years were 69.7% and 44.8% respectively (p = 0.000). Recipient age ≥ 40 years, advanced stage and Busulfan-Fludarabine(BuFlu) conditioning regimen were identified as major risk factors for aGVHD. Recipient age ≥ 40 years, BuFlu conditioning regimens, female donor/male recipient and prior aGVHD were associated with cGVHD. Despite lower RM (relapse mortality), patients with grade 2–4 aGVHD had higher NRM and worse OS and LFS compared to patients with grade 0–1 aGVHD. In contrast, patients with cGVHD had better OS and LFS and lower RM compared to patients without cGVHD.

**Conclusion:**

The novel GVHD regimen decreased the risk for aGVHD by 42% without improving the risk for cGVHD compared to historical controls. Development of aGVHD was associated with worse OS and LFS as well as higher NRM. In contrast, cGVHD was associated with improved OS and LFS likely attributed to a GVL effect.

## Introduction

Despite the use of prophylaxis regimens, Graft-versus-host disease (GVHD) remains a major cause for mortality and morbidity with allogeneic hematopoietic stem cell \transplantations (allo-HSCT). It is also the primary cause of death in 16% and 18% of deaths after HLA-match sibling and unrelated donor allo-HSCT respectively [1]. A combination consisting of a calcineurin inhibitor (CNI), cyclosporine or tacrolimus, and either methotrexate, mycophenolate mofetil (MMF), or sirolimus are considered to be the standard prophylaxis regimens. However, review of US and European literature indicates that acute GVHD (aGVHD) still occurs in 35% to 65% of BMT patients receiving human leukocyte antigen (HLA)–matched sibling transplants, and even more frequently in unrelated donor transplant recipients [2]-[6]. Analysis of Chinese transplant registries as well as relevant Chinese publications calculate the overall incidence for grade 2–4 aGVHD at approximately 40% [7]-[9]. Thus, improved prophylactic approaches are needed. Most strategies employed to reducing both aGVHD and chronic GVHD (cGVHD) (e.g. T-cell depletion) have significant drawbacks as they are offset by high rates of graft failure, malignancy relapse, infections, and Epstein-Barr virus-associated lymphoproliferative disorders [10]-[12]. For patients with hematologic malignancies, “standard of care GVHD prophylaxis” seems to have struck a reasonable balance between preventing undesirable graft-versus-host reactions and retaining desirable graft-versus-tumor effects [13]. The risk for developing GVHD depends on various factors which are determined by the patient, disease characteristics as well as by the graft, its processing, and the transplant procedure/conditioning regimen employed. Thus far, no trials have been conducted where GVHD prophylaxis has been individually stratified to the probability of GVHD occurrence or disease relapse.

We have previously described a combination prophylaxis regimen consisting of cyclosporine A (CsA), methotrexate (MTX) and a low-dose, short-course mycophenolate mofetil (MMF) (0.5 daily for 30 days) in a single institution trial for a cohort of 100 patients with hematologic malignancies who underwent HLA-matched sibling allo-HSCT. The rationale behind a regimen designed with a short course of MMF was to primarily improve aGVHD without substantially impacting the incidence of cGVHD because of the associated beneficial GVL effect. Although cGVHD is an undesired complication of BMT, we hypothesized that this strategy would lead to reduced leukemia relapse to an extent which would result in an overall survival net benefit across all patients. We did indeed report a substantial decrease in the risk for aGVHD in our initial study [14]. In order to confirm the effectiveness of this new GVHD prophylaxis regimen, the Chinese Bone Marrow Transplant Cooperative Group (CBMTCG) initiated a prospective, open-label, multicenter clinical trial using this prophylaxis regimen in 508 patients. Furthermore, we analyzed additional risk factors for GVHD in this population consisting entirely of Chinese patients.

## Patients and methods

### Patient eligibility

The trial was designed as a prospective, open-label, multicenter clinical protocol and was conducted by the Chinese Bone Marrow Transplant Cooperative Group (CBMTCG), a cooperative transplant group consisting of seven Chinese transplant centers: the Peking University Institute of Hematology, n = 264; the First Affiliated Hospital of Guangxi Medical University, n = 81; the First Affiliated Hospital of Xinjiang Medical University, n = 50; Changzheng Hospital, the Second Military Medical University, n = 10; Kunming General Hospital of Chengdu Command, n = 55; Nanfang Hospital Southern Medical University, n = 28; First Affiliated Hospital Chongqing Medical University, n = 20. Patients with hematologic malignancies in need of an allo-HSCT who had an HLA-identical sibling donor were eligible. Additional eligibility criteria included: 1) Age:15 to 65 years old; 2) Medically suitable to tolerate a myeloablative (MA) conditioning regimen; 3) Eastern Cooperative Oncology Group (ECOG) performance status ≤ 2; 4) Bilirubin ≤ 2 mg/dL, 5) creatinine < 1.5 times the upper limit of normal, 6) preserved heart and lung function; 7) Negative infectious evaluation (viral, bacterial and fungal). Between August 2007 and October 2010, 508 patients were enrolled and completed treatment. The protocol was approved by the Institutional Review Board of each center, and prior treatment written informed consent was obtained from both patients and donors. All participating institutions and investigators had subscribed to the principles and conduct of the WMA Declaration of Helsinki - Ethical Principles for Medical Research Involving Human Subjects. Patient characteristics are summarized in Table 1.

**Table 1:** Patients and transplantation characteristics

**Variables**	**N**	**%**
Patients	508	
Male	294	57.9
Female	214	42.1
Median age, years (range)	36 (15–62)	
15-39	313	61.6
≥40	195	38.4
Disease		
AML	231	45.5
CR	188	37.0
PR	2	0.4
NR	41	8.1
ALL	86	16.9
CR	80	15.7
NR	6	1.2
CML	156	30.7
CP	127	25.0
AP	10	2.0
BC	19	3.7
MDS-RAEB	35	6.9
Disease stage		
Non-advanced	441	86.8
Advanced	67	13.2
Donor-recipient gender		
Female–male	140	27.6
other	368	72.4
Stem cell sources		
PB + BM	250	49.2
PB	258	50.8
ABO blood group status		
Match	305	60.0
Mismatch	203	40.0
MMF Dose		
0.5 g/d	151	29.7
1.0 g/d	357	70.3
Conditioning regimens		
BuCy	347	68.3
TBICy	26	5.1
BuFlu	135	26.6

AML, acute myeloid leukemia; CR, complete remission; PR, partial remission; NR, nonremission; ALL, acute lymphocytic leukemia; CML, chronic myelogenous leukemia; CP, chronic phase; AP, accelerated phase; BC, blast crisis; MDS, myelodysplastic syndrome; RAEB, refractory anemia with excess blasts; Non-advanced stage, including acute leukemia (AL) in CR, CML in CP and AP, MDS-RAEB, Advanced stage, including AL in NR and CML in BC, PB, peripheral blood; BM, bone marrow; TBI, total body irradiation; Cy, cyclophosphamide; Bu, busulfan; MMF, mycophenolate mofetil, Flu, fludarabin.

### Conditioning regimen

The protocol permitted use of three myeloablative conditioning regimens at investigators discretion: 1) BuCy: Busulfan (12–16 mg/kg) and cyclophosphamide (120 mg/kg) was given to 347 patients. 2) BuFlu: Busulfan (12–16 mg/kg) combined with Fludarabine (250 mg or 150 mg/m2) was given to 134 patients and Fludarabine (250 mg) combined with Melphalan (140 mg/m2) was given to one patient. 3) TBICy: TBI (7.7-10.0Gy) and cyclophosphamide (120 mg/kg) was given to 26 patients.

#### Procurement of hematopoietic stem cells

The protocol permitted use of two sources of hematopoietic stem cells at investigators discretion: 1) Peripheral Blood (PB): 258 patients were grafted with human granulocyte colony-stimulating factor (rHuG-CSF)–mobilized peripheral blood stem cells (PBSC).Donors were treated with G-CSF at a dose of 5–10 ug/kg/d subcutaneously for 4 consecutive days starting 5 days before leukapheresis with a target CD34 cell number of at least 4 × 10^6^ CD34cells per kilogram of recipient weight. The median numbers of mononuclear cells (MNC) and CD34 cells infused were 9.11 (2.93-17.6) × 10^8^/kg and 4.25 (1.15-16.6) × 10^6^/kg, respectively. 2) PB + BM: 250 patients were grafted with a combination PBSC and bone marrow stem cells (BMSC). PBSC were harvested after donors were treated with G-CSF at a dose of 5–10 ug/kg/d subcutaneously for 4 consecutive days and bone marrow was harvested on the following day by standard technique using general anesthesia. The ratio of CD34 cells numbers of PB to BM was 2–4:1. The median numbers of MNCs and CD34 cells infused were 7.48(3.14-12.53) × 10^8^/kg and 2.34 (0.40-7.45) × 10^6^/kg, respectively. This combination grafting procedure is commonly used in China and based on publications previously describing improved outcomes [15].

### GVHD prophylaxis

All of the transplant recipients received CsA, MTX, and a low-dose, short-course MMF. The dosage of CsA was 2.5-3 mg/kg/d, i.v., and CsA was administered from day 1 before transplantation until recovery of bowel function. At that point, the patient was switched to oral CsA. Serum CsA concentration was monitored, and the dosage was adjusted to achieve serum concentrations ranging between 150–300 ng/ml. MMF was administered orally as doses of 0.5-1.0 g/d from day 1 before transplantation to day 30 after transplantation. The dose was assigned by weight, with patients up to and below 60 kg receiving 0.5 mg and patients above 60 kg receiving 1.0 mg. MTX was administered i.v. at doses of 15 mg/m^2^ on day 1 and 10 mg/m^2^ on days 3, 6 and 11. Fifty-nine patients omitted the 4th dose of MTX. First-line therapy of clinically significant aGVHD consisted of methylprednisolone (MP) 1 to 2 mg/kg/d. Patients whose GVHD was refractory to steroid therapy could receive secondary therapy such as tacrolimus, CD25 antibody or other therapies at investigator discretion.

### Definitions and assessments

Disease status at transplant was classified as “non-advanced stage” or “advanced stage.” The patients were categorized as “ non-advanced stage” if they were in complete remission (CR) from acute leukemia (AL) regardless of cytogenetics, chronic myelogenous leukemia (CML) in chronic phase (CP) or accelerated phase (AP), and myelodysplastic syndrome(MDS) with a blast count <20%. “Advanced stage” was defined as AL not in remission (NR) or CML in blast crisis (BC). Neutrophil engraftment was defined as the first day of an absolute neutrophil count (ANC) of 0.5 × 10^9^/L or more for 3 consecutive days, and platelet engraftment was defined as the first day of platelets ≥ 20 × 10^9^/L for 7 consecutive days without transfusion. Primary engraftment failure was defined as the absence of donor-derived myeloid cells at day 60 in patients surviving beyond day 28 after transplantation. Chronic GVHD was evaluated in patients who survived for greater than 100 days and had a sustained engraftment. Acute and chronic GVHD were defined according to published standard criteria [16],[17]. Relapse was defined as evidence for the presence of morphological disease in peripheral blood, marrow, or extramedullary sites. Leukemia-free survival (LFS) was defined as continuous CR at the last follow-up.

### Statistical methods

For our Chinese population treated in China, historically grade 2–4 aGVHD is reported to be around 40% [7]-[9]. Our primary endpoint was to improve the grade 2–4 aGVHD incidence by 25% over historical control (reduce to 30%). Under the assumptions of 90% power and a two sided error rate of 0.05 we calculated the trial a size of 477 patients. By estimating a 5% dropout rate, we planned our sample size at 500 patients. Achieving the primary endpoint was calculated to be associated with a HR of 0.75.

Cumulative incidences were estimated for engraftment, aGVHD, cGVHD, non-relapse mortality (NRM), relapse mortality (RM) and relapse in order to evaluate competing risks. The competing risk for engraftment was death without engraftment; the competing risk for GVHD was death without GVHD and graft rejection; relapse was a competing risk for NRM; and NRM was a competing risk for relapse. The time of GVHD occurrence was defined from day 1 after graft infusion to the onset of any grade of GVHD; aGVHD was censored at day 100 after HSCT and cGVHD was censored at the last follow-up visit. The worst stage of the GVHD was assessed as the degree of the GVHD reported. The probabilities of overall survival (OS) and LFS were estimated by the Kaplan-Meier method [18]. Potential prognostic factors were evaluated in univariate analyses by the log-rank test, with a P-value of less than 0.05 being considered statistically significant. The demographics of patients and donors, the underlying disease, disease status, conditioning regimens, the source of the graft, MMF doses and other pre-transplant parameters were included in the univariate analyses. In the multivariate analysis, all of the factors found to influence the outcomes in the univariate analysis with a P < 0.1 were included into a Cox proportional hazard model using a forward: conditional method (SAS version 8.2, SAS Institute, Cary, NC, and S Plus 2000, Mathsoft, Seattle, WA). Data cut off for survival follow-up was October 31, 2011. The median follow-up time was 22.8 months.

## Results

### Engraftment

503 (99.0%) patients achieved sustained myeloid engraftment. The median time to reaching an ANC above 0.5 × 10^9^ cells/L was 14 (7–24) days. During the follow-up period, 496 patients (97.6%) exhibited platelet engraftment, and the median time to reach a platelet count above 20 × 10^9^ cells/ L was 13 (6–124) days.

### Graft-versus-host disease

**Figure 1:**
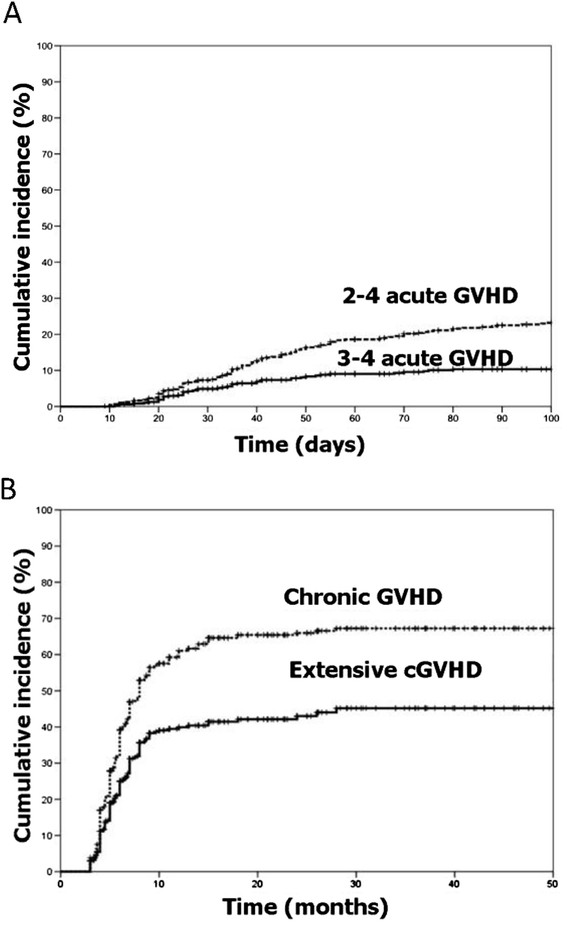
**Cumulative incidences of aGVHD (Figure**1**-A) and cGVHD (Figure**1**-B) in 508 patients who underwent sibling identical allo-HSCT and CSA/MTX/MMF GVHD prophylaxis.**

At 100 days after transplantation, the cumulative incidence was 23.2% (95% CI, 21.2%-25.2%) for grade 2 to 4 aGVHD, and 10.3% (95% CI, 9.8%-11.8%) for grade 3 to 4 aGVHD (Figure 1-A). The cumulative incidence was 67.4% (95% CI, 64.9%-69.9%) for total cGVHD and 45.1% (95% CI, 42.0%-48.2%) for extensive cGVHD at 2 years after transplantation (Figure 1-B).

### Analysis of risk factors for aGVHD

The risk factors for aGVHD determined by univariate analysis are listed in Table 2. By univariate analysis, patient and donor age ≥ 40 years old, advanced stage as well as BuFlu conditioning regimen were associated with grade 2 to 4 aGVHD incidence. Advanced stage, BuFlu conditioning regimen were associated with the risk of grade 3 to 4 aGVHD. Donor-recipient sex match, donor-recipient blood type match, MMF dose and CML were not associated with grade 2 to 4 or grade 3 to 4 aGVHD incidences. Because in our study all donors were siblings, the age range for patients and donors remained relatively narrow (r = 0.797, p = 0.000). For recipients younger than 40 years of age, no impact of donor age on the incidence or severity of aGVHD was observed. Older patients (≥40 years) had higher odds to experience grade 2–4 aGVHD than the younger patients (<40 years) (30.0% vs 18.5%, p = 0.01).Patients transplanted at advanced stage had significantly higher odds of developing grade 2–4 aGVHD (30.8% vs 21.7%, p = 0.045) and grade 3–4 aGVHD (21.8% vs 8.5%, p = 0.000) when compared to patients who were transplanted at non-advanced stage. By multivariate analysis, patient age ≥ 40 years and BuFlu conditioning regimen were associated with grade 2 to 4 aGVHD incidence. Advanced stage and BuFlu conditioning regimen were associated with the risk of grade 3 to 4 aGVHD (Table 3).

**Table 2:** Risk factors for acute graft-versus-host disease determined by univariate analysis

**Variable**	**N**	**Grade 2–4 aGVHD**			**Grade 3–4 aGVHD**		
		**HR**	**95% CI**	**P**	**HR**	**95% CI**	**P**
Age for recipients,years							
<40	305	1.00			1.00		
≥40	189	1.62	1.11-2.36	0.013	1.14	0.64-2.06	0.656
Age for donor, years							
<40	306	1.00			1.00		
≥40	188	1.50	1.01-2.24	0.046	0.90	0.48-1.68	0.732
Age for donor and recipients, years							
Recipients < 40 and donor < 40	260	1.00			1.00		
Recipients < 40 and donor ≥ 40	56	1.08	0.54-2.16	0.821	0.58	0.17-1.92	0.370
Recipients ≥ 40 and donor < 40	46	1.23	0.60-2.52	0.578	0.97	0.34-2.80	0.953
Recipients ≥ 40 and donor ≥ 40	132	1.77	1.13-2.77	0.013	1.03	0.51-2.08	0.929
Donor-recipient gender							
Other	354	1.00			1.00		
Female → Male	140	1.13	0.74-1.71	0.574	1.19	0.64-2.21	0.575
Stem cell source							
BM + PB	240	1.00			1.00		
PB	254	1.17	0.80-1.70	0.430	1.54	0.85-2.78	0.155
Leukemia type							
AL	305	1.00			1.00		
CML	155	0.71	0.46-1.12	0.142	0.76	0.39-1.47	0.411
Conditioning regimen							
BuCy	341	1.00			1.00		
TBICy	24	1.75	0.80-3.81	0.160	2.20	0.66-7.36	0.199
BuFlu	128	1.63	1.08-2.46	0.020	2.86	1.57-5.20	0.001
Donor-recipient blood type							
Match	306	1.00			1.00		
Mis-match	188	0.93	0.62-1.40	0.730	0.85	0.47-1.58	0.611
Disease stage							
Non-advanced	429	1.00			1.00		
Advanced	65	1.67	1.01-2.77	0.047	3.17	1.67-6.03	0.000
MMF dose							
0.5 g/d	128	1.00			1.00		
1.0 g/d	366	1.39	0.87-2.22	0.168	1.01	0.52-1.94	0.983

aGVHD, acute graft-versus-host disease; AL, acute leukemia; CML, chronic myelogenous leukemia; BM, bone marrow; BP, peripheral blood; TBI, total body irradiation; Cy, cyclophosphamide; Bu, busulfan; MMF, mycophenolate mofetil; Flu, fludarabin; Non-advanced stage, including acute leukemia (AL) in CR, CML in CP and AP, MDS-RAEB; Advanced stage, including AL in NR and CML in BC.

**Table 3:** Multi-factor analysis of risk factors for acute graft-versus-host disease determined

**Variable**	**N**	**Grade 2–4 aGVHD**			**Grade 3–4 aGVHD**		
		**HR**	**95% CI**	**P**	**HR**	**95% CI**	**P**
Age for recipients,years							
<40	305	1.00			1.00		
≥40	189	1.65	1.13-2.41	0.010	1.19	0.66-2.14	0.565
Conditioning regimen							
BuCy	341	1.00			1.00		
TBICy	24	1.67	0.76-3.71	0.205	1.71	0.50-4.61	0.395
BuFlu	128	1.58	1.04-2.39	0.033	2.49	1.35-4.61	0.004
Disease stage							
Non-advanced	429	1.00			1.00		
Advanced	65	1.44	0.85-2.43	0.172	2.45	1.26-4.78	0.009

aGVHD, acute graft-versus-host disease; Bu, busulfan; Cy, cyclophosphamide; TBI, total body irradiation; Flu, fludarabin; Non-advanced stage, including acute leukemia (AL) in CR, CML in CP and AP, MDS-RAEB; Advanced stage, including AL in NR and CML in BC.

### Analysis of risk factors for cGVHD

Risk factors for cGVHD determined by univariate analysis are listed in Table 4. By univariate analysis, patient age ≥ 40 years old, female donor/male recipient, BuFlu regimen for conditioning and prior aGVHD were the risk factors for both cGVHD and extensive cGVHD. Univariate analysis did not identify donor age, advanced stage, donor-recipient blood type match or CML to be risk factors for cGVHD and extensive cGVHD. Patients ≥ 40 years old were found to have higher odds for developing cGVHD and extensive cGVHD than younger patients (<40 years) (75.9% vs 62.3%, p = 0.016) and (55.9% vs 38.6%, p = 0.007).No significant differences for cGVHD and extensive cGVHD were found for patients transplanted at non-advanced stage or advanced stage (67.3% vs 62.5%, p = 0.374) and (45.6% vs 33.0%, p = 0.888). Multivariate analysis confirmed female donor/male recipient, BuFlu regimen for conditioning and prior aGVHD as risk factor for cGVHD and extensive cGVHD (Table 5).

**Table 4:** Univariate analysis of risk factors for chronic graft-versus-host disease

**Variable**	**N**	**cGVHD**			**Extensive cGVHD**		
		**HR**	**95% CI**	***P***	**HR**	**95% CI**	***P***
Age for recipients, years							
<40	286	1.00			1.00		
≥40	180	1.34	1.06-1.70	0.016	1.54	1.12-2.11	0.007
Age for donor, years							
<40	288	1.00			1.00		
≥40	178	1.10	0.848-1.43	0.473	1.362	0.971-1.912	0.074
Age for donor and recipients, years							
recipients < 40 and donor < 40	243	1.00			1.00		
recipients < 40 and donor ≥ 40	52	0.72	0.46-1.15	0.174	0.90	0.49-1.64	0.729
recipients ≥ 40 and donor < 40	47	1.24	0.82-1.88	0.314	1.46	0.84-2.53	0.179
recipients ≥ 40 and donor ≥ 40	124	1.38	1.03-1.85	0.034	1.76	1.20-2.59	0.004
Donor-recipient gender							
Other	336	1.00			1.00		
Female → Male	130	1.48	1.15-1.90	0.002	1.92	1.39-2.65	0.000
Stem cell source							
BM + PB	241	1.00			1.00		
PB	225	0.51	0.40-0.65	0.000	0.30	0.21-0.43	0.000
Leukemia type							
AL	287	1.00			1.00		
CML	145	1.04	0.80-1.34	0.778	0.94	0.66-1.35	0.747
Conditioning regimen							
BuCy	326	1.00			1.00		
TBICy	23	0.66	0.34-1.29	0.226	0.56	0.21-1.54	0.264
BuFlu	116	1.49	1.15-1.94	0.003	1.96	1.41-2.73	0.000
Donor-recipient blood type							
Match	273	1.00			1.00		
Mis-match	193	1.14	0.89-1.45	0.301	1.18	0.85-1.60	0.317
Disease Stage							
Non-advanced	415	1.00			1.00		
Advanced	51	1.19	0.80-1.78	0.389	0.96	0.54-1.70	0.890
aGVHD							
No	332	1.00			1.00		
Yes	134	1.82	1.42-2.34	0.000	2.14	1.55-2.96	0.000

aGVHD, acute graft-versus-host disease; cGVHD, chronic graft-versus-host disease; BM, bone marrow; BP, peripheral blood; AL, acute leukemia; CML, chronic myelogenous leukemia; TBI, total body irradiation; Cy, cyclophosphamide; Bu, busulfan; MMF, mycophenolate mofetil; Flu, fludarabin; Non-advanced stage, including acute leukemia(AL) in CR,CML in CP and AP, MDS-RAEB; Advanced stage, including AL in NR and CML in BC.

**Table 5:** Multi-factor analysis of risk factors for chronic graft-versus-host disease

**Variable**	**N**	**cGVHD**			**Extensive cGVHD**		
		**HR**	**95% CI**	**P**	**HR**	**95% CI**	**P**
Age for recipients, years							
<40	286	1.00			1.00		
≥40	180	1.24	0.96-1.59	0.093	1.35	0.97-1.87	0.072
Donor-recipient gender							
Other	336	1.00			1.00		
Female → Male	130	1.35	1.04-1.75	0.026	1.67	1.20-2.34	0.002
Conditioning regimen							
BuCy	326	1.00			1.00		
TBICy	23	0.62	0.30-1.24	0.175	0.91	0.20-1.50	0.244
BuFlu	116	1.38	1.05-1.81	0.020	1.69	1.20-2.39	0.003
aGVHD							
No	332	1.00			1.00		
Yes	134	1.73	1.34-2.23	0.000	1.94	1.39-2.69	0.000

aGVHD, acute graft-versus-host disease; cGVHD, chronic graft-versus-host disease; Bu, busulfan; Cy, cyclophosphamide; TBI, total body irradiation; Flu, fludarabin.

### Survival, relapse and long-term follow-up

As of Apr 30, 2011, 377 patients were alive following transplantation, with a median survival time of 22.8 m (range: 6-52 m). Probabilities for OS and LFS in all patients at 2 years were 68.0% (95% CI =63.3%-75.8%) and 66.0% (95% CI =63.3%-75.8%) respectively. Probabilities for OS in patients transplanted at non-advanced stage and advanced stage at 2 years were 71.4% (95% CI =65.9%-77.0%) and 41.8% (95% CI =20.9%-62.6%) (p = 0.000), respectively. The probabilities of LFS in non-advanced stage and advanced stage patients at 2 years were 69.7% (95% CI =64.7%-74.8%) and 44.8% (95% CI =30.7%-58.9%)(p = 0.000), respectively.

Sixty-nine patients relapsed after transplantation, reaching a cumulative relapse of 17.7% (95% CI, 15.6%-19.8%) at 2 years. The NRM rate was 4.3% (95% CI = 3.4% - 5.2%) and 18.4% (95% CI = 16.3%-20.5%) at 100 days and 2 years, respectively. 53 out of 131 patients died of leukemia relapse. Seventy-eight patients died from other causes than relapse. The most frequent cause of death was infection, specifically, pneumonia. Severe GVHD was determined as cause of death in nineteen patients.

### Comparison of outcomes in patients between with and without acute and/or chronic GVHD

In order to analyze the impact of GVHD on the overall clinical outcomes of our patients, we compared outcomes for patients with and without acute and/or chronic GVHD (Table 6). Although the patients with grade 2–4 aGVHD had lower RM, they had higher NRM and worse OS and LFS when compared with the patients grade 0–1 aGVHD. In contrast, patients with cGVHD had improved OS and LFS and lower RM compared to patients without cGVHD (Table 6).

**Table 6:** Comparison of outcomes in patients between with and without acute and/or chronic GVHD

	**0-1 aGVHD% (95% CI) n = 386**	**2-4 aGVHD% (95% CI) n = 108**	***P***	**Non- cGVHD% (95% CI) n = 189**	**cGVHD% (95% CI) n = 277**	***P***
NRM	14.3% (CI = 10.7%-19.3%)	32.0% (CI = 23.4%-43.9%)	0.000	15.7% (CI = 10.6%-23.2%)	12.1% (CI = 8.3%-17.6%)	0.194
RM	20.6% (CI = 16.2%-26.3%)	8.6% (CI = 4.2%-17.8%)	0.036	25.4% (CI = 19.0%-34.0%)	11.6% (CI = 7.6%-17.5%)	0.000
OS	69.4% (CI = 63.2%-75.6%)	62.7% (CI = 52.6%-72.9%)	0.009	62.0% (CI = 53.0%-71.1%)	80.0% (CI = 73.5%-86.4%)	0.000
LFS	68.0% (CI = 62.5%-73.5%)	61.0% (CI = 50.8%-71.1%)	0.047	61.8% (CI = 53.9%-69.7%)	77.4% (CI = 71.5%-83.3%)	0.000

aGVHD, acute graft-versus-host disease; cGVHD, chronic graft-versus-host disease; NRM, non-relapse mortality; RM, relapse mortality; LFS, leukemia free survival; OS, overall survival.

## Discussion

Although GVHD has been recognized more than fifty years as a complication of allo-HSCT, current prophylactic therapies remain insufficient and a high medical need to improve outcomes remains [19]-[21]. Thus far, no significant progress has been made in developing novel aGVHD regimens and most approaches are not improved over historical results. Although Devine et al. reported in their study grade 2–4 aGVHD in only 22.4% and extensive cGVHD of 6.8%, this was offset by only 58% 3 years DFS in patients transplanted in AML-CR1. Likely, this was related to graft T cell depletion (TCD) which was part of the GVHD prophylaxis in this study [22]. Compared to historic transplant results derived from China, European and US centers, this multicenter trial demonstrates a substantial decrease of aGVHD in HLA-matched sibling allo-HSCT without increasing disease relapse or adversely impacting survival in standard risk patients, Our outcomes rather compare to results published by Tanimoto TE, et al. from Japan ( Table 7) [2]-[5],[23]-[25].

**Table 7:** Comparative summary of GVHD frequencies and clinical outcomes in selected studies for HLA-match sibling myeloablative hsct

	**Our results n = 508**	**Tanimoto et al. [**[5]**] n = 509**	**Couban et al. [**[3]**] n = 109**	**Schmitz et al. [**[4]**] n = 163**	**Bensinger et al. [**[2]**] n = 81**	**Yang et al. [**[24]**] n = 68**	**Ringde′n et al.[**[25]**] n = 1887**
GVHD prophylaxis regimen	MMF + CSA + MTX	CSA + MTX	CSA + MTX	CSA + MTX	CSA + MTX	CSA + MTX	CSA ± MTX
2-4 aGVHD(%)	23	32 ~ 37	44	52	64	46	37 ~ 46
3-4 aGVHD(%)	10	9 ~ 18	26	28	15	9	26 ~ 38
cGVHD(%)	67	42 ~ 62	85	67	46	45	26 ~ 38
extensive cGVHD(%)	45	27 ~ 42	40	ns	37	ns	ns
OS(%)	71*	74*	68*	65*	66*	68	ns
LFS(%)	70*	65 ~ 68*	ns	ns	65	62	33 ~ 49
NRM(%)	18	16 ~ 19	ns	ns	21	ns	25 ~ 38

*Standard risk patients.

aGVHD, acute graft-versus-host disease; cGVHD, chronic graft-versus-host disease; HLA, human leukocyte antigen; HCST, hematopoietic stem cell transplantation; NRM, non-relapse mortality; LFS, leukemia free survival; OS, overall survival; CsA, cyclosporine; MTX, methotrexate; MMF, mycophenolate mofetil.

Several studies have identified risk factors for GVHD over the past 3 decades [21],[26]-[33]. Gale et al. analyzed data of 2036 recipients of HLA-identical sibling transplants between 1978 and 1985 within the IBMTR. They found donor/ recipient sex-match, patient age ≥40 years and lack of GVHD prophylaxis to be associated with moderate to severe GVHD [26]. Hahn et al. analyzed IBMTR data of 1,960 adults after sibling HLA-identical myeloablative transplant performed between 1995 and 2002. They reported risk factors for grade 2 to 4 acute GVHD to be age 40 and older, use of total body irradiation (TBI), grafting with mobilized blood cells , CML versus AML/ALL, white/Black versus Asian/Hispanic race (recipient), Karnofsky performance score less than 90 versus 90 to 100 ,and recipient/donor cytomegalovirus-seronegative status [21]. Another study by Flowers et al. analyzed 2941 adult and pediatric patients with both related and unrelated HLA –matched allo-HSCT performed between 1992 and 2005 in Seattle [33]. Risk factors for developing grade 2 to 4 acute GVHD were unrelated donors, use of TBI, lack of ATG utilization, female donor/male recipient and the underlying diagnosis of CML. Concurring with most other previous reports we determined in our study the main risk factors for both aGVHD and cGVHD to be age ≥ 40 years [21],[26] . In contrast to most other reports, we found that neither donor age, nor donor/recipient sex match, blood type match or CML enhanced the likelihood for aGVHD, however, donor/recipient sex match was associated with an increased risk of cGVHD. Whether donor age impacts the risk of GVHD in our patient population needs to be further studied. Kollman et al. reported that donor age was associated with aGVHD and cGVHD in unrelated donor HSCT [34]. Because all donors were siblings, the age range for patients and donors in our study remained in a relatively narrow range, and therefore our sample size was insufficient for certain subset analysis. However, we did not observe in recipients younger than 40 years of age any impact of the donor age on either aGVHD or cGVHD. Our data suggest that the BuFlu conditioning regimen significantly increases the incidence rates for both aGVHD and cGVHD when compared with the BuCy regimen. Such association has previously not been reported in other studies and therefore these results need to be interpreted with caution. In fact, Chae et al. reported the opposite, that the BuFlu regimen decreased both incidence rates of aGVHD as well as cGVHD when compared with BuCy regimen [35]. On the other hand, Lee [36] and Liu [37] found no significant differences for the incidence rates of aGVHD and cGVHD between BuCy and BuFlu conditioning regimens in two randomized trials.

We observed cumulative incidences of cGVHD and extensive cGVHD at 2 years at 67.4% and 45.1%, respectively. Compared to some historic studies (Table 7), at least this aspect of our study could be interpreted as lack of improvement over current standards. The cumulative incidences for cGVHD and extensive cGVHD were slightly lower with 53.3% and 28.2% reported in our prior study [14]. We explain this relatively high incidence of cGVHD in our study with the fact that MMF was only given for 30 days. However, this short course was deliberately chosen to maintain some cGVHD in an effort to maximize the GVL effect [38]. Another explanation for this finding might be that all of our patients’ grafts were PBSCT based and consisted either of PBSCT alone or PBCST + BM. Therefore higher rates of cGVHD are expected compared to studies using pure BMT grafts [15]. For instance, Wang Y et al. reported the incidence for cGVHD at 50% for haploBMT [39]. There is no study providing conclusive evidence regarding any differences for the incidence of GVHD between Chinese and Caucasian patients. However, a key factor determining development of cGVHD is the duration of immunosuppression. The design of our regimen prescribes only a short course (30 days). Although the regimen is effective in preventing aGVHD, the remaining immunosuppression after MMF discontinuation may not have been sufficient enough and thus resulted in the observed cGVHD increase.

Although we found in our study grade 2–4 aGVHD to be associated with lower rates of relapse mortality, this also resulted in worse OS and LFS mainly due to increased NRM. In contrast, we found that cGVHD was associated with better OS and LFS outcomes which we explain with concurrently decreased rates of relapse mortality (Table 6). Nevertheless, these data have to be interpreted with caution because 30.7% of our patients had a diagnosis of CML, a disease known to be more susceptible to GVL manipulations and the follow up has been only 2 years. Moreover, other studies showed worse survival for both cGVHD and aGVHD, despite a favorable association of cGVHD on disease relapse (AML and MDS) [40]. Clearly, additional data need to be generated to fine-tune GVHD regimens in order to maximize the therapeutic benefit and find the optimal balance between aGVHD, cGVHD and GVL.

## Conclusion

In summary, although due to the study design a direct comparison of the low dose short course MMF containing regimen to CsA/MTX alone for GVHD prophylaxis could not be performed, we demonstrated a substantial decrease for the risk of aGVHD development. In contrast, the incidence for cGVHD could not be improved when compared to historical results in Chinese patients. Our results also suggest that Chinese patients may have slight variations in risk factors for developing GVHD.

## Abbreviations

allo-HCST: Allogeneic hematopoietic cell transplantation

GVHD: Graft-versus-host disease

aGVHD: Acute GVHD

cGVHD: Chronic GVHD

CsA: Cyclosporine

MTX: Methotrexate

MMF: Mycophenolate mofetil

HLA: Human leukocyte antigen

LFS: Leukemia free survival

OS: Overall survival

CR: Complete remission

TBI: Total body irradiation

G-CSF: Granulocyte colony-stimulating factor

MNC: Mononuclear cells

NRM: Non-relapse mortality

RM: Relapse mortality

## Competing interests

The authors declare that they have no competing interests.

## Authors’ contributions

YRL and YHC designed the research, interpreted the data and wrote the manuscript; DMH, MJ, QL, LL, JH, PS, QCL, ZMZ and KYL performed the study and contributed to writing the manuscript; XJH is the principal investigator, designed the research, interpreted the data, and wrote the manuscript. All authors read and approved the final manuscript.
